# Identifying common prognostic factors in genomic cancer studies: A novel index for censored outcomes

**DOI:** 10.1186/1471-2105-11-150

**Published:** 2010-03-24

**Authors:** Sigrid Rouam, Thierry Moreau, Philippe Broët

**Affiliations:** 1 Computational and Mathematical Biology, Genome Institute of Singapore, Singapore 138672, Singapore; 2 Univ Paris-Sud, JE2492, Villejuif, F-94807 France; 3 Inserm, U780, Villejuif, F-94807 France; Univ Paris-Sud, Villejuif, F-94807 France

## Abstract

**Background:**

With the growing number of public repositories for high-throughput genomic data, it is of great interest to combine the results produced by independent research groups. Such a combination allows the identification of common genomic factors across multiple cancer types and provides new insights into the disease process. In the framework of the proportional hazards model, classical procedures, which consist of ranking genes according to the estimated hazard ratio or the p-value obtained from a test statistic of no association between survival and gene expression level, are not suitable for gene selection across multiple genomic datasets with different sample sizes. We propose a novel index for identifying genes with a common effect across heterogeneous genomic studies designed to remain stable whatever the sample size and which has a straightforward interpretation in terms of the percentage of separability between patients according to their survival times and gene expression measurements.

**Results:**

The simulations results show that the proposed index is not substantially affected by the sample size of the study and the censoring. They also show that its separability performance is higher than indices of predictive accuracy relying on the likelihood function. A simulated example illustrates the good operating characteristics of our index. In addition, we demonstrate that it is linked to the score statistic and possesses a biologically relevant interpretation.

The practical use of the index is illustrated for identifying genes with common effects across eight independent genomic cancer studies of different sample sizes. The meta-selection allows the identification of four genes (*ESPL1*, *KIF4A*, *HJURP*, *LRIG1*) that are biologically relevant to the carcinogenesis process and have a prognostic impact on survival outcome across various solid tumors.

**Conclusion:**

The proposed index is a promising tool for identifying factors having a prognostic impact across a collection of heterogeneous genomic datasets of various sizes.

## Background

In clinical cancer research, recent advances in genome-wide technologies have enabled researchers to identify large-scale genomic changes having a potential prognostic impact on time-to-event outcomes. The growing number of public repositories for high-throughput genomic data facilitates the retrieval and combination of various datasets produced by independent research groups (for a few: *GEO *[1], *Oncomine *[2], *ArrayExpress *[3]). These databases potentially represent valuable resources for identifying genomic factors that have a common prognostic impact on clinical outcomes (e.g. time to local or distant recurrence) across multiple cancer types. However, the joint analysis of these heterogeneous datasets is difficult due to the fact that they are usually of varying sample size, investigate different survival outcomes or are related to different tumors entities. In this context, defining a procedure for identifying common genomic risk factors across multiple heterogeneous datasets is a promising but very challenging task. In recent years, several authors [4-7] have proposed meta-profiling methods for class comparison, designed to identify common transcriptional features of the tumoral process (normal versus tumor state).

In the framework of the widely used Cox model [8] for analyzing possibly censored time-to-event or survival data, different procedures for feature selection across multiple gene expression datasets can be defined. Basically, each gene expression measurement is included in a simple Cox model, giving rise to an estimation of the corresponding hazard ratio and to a statistic for testing the null hypothesis of no association between survival outcome and gene expression changes. Simple procedures, frequently used in practice, consist of ranking the genes in each dataset from the highest (or lowest) value to the lowest (or highest) value according to either the estimated hazard ratio or quantities derived from the test statistic (e.g. p-value), and finally to select those that appear at the intersection of the lists using a defined thresholding procedure [9]. However, these approaches suffer serious drawbacks that are mostly related to the chosen selection criteria. Choosing the estimated hazard ratio clearly ignores the variability of the data, while the choice of quantities derived from test statistics leads to emphasize large datasets, since it is well known that every test statistic increases with the sample size.

In meta-selection of heterogeneous genomic datasets, taking into account both the magnitude of the prognostic impact of factors and the variability of the data without being highly dependent on the sample size is likely to be more biologically relevant. Addressing this issue led us to propose a novel index designed for genomic survival analysis that provides information about the capability of a genomic factor to separate patients according to their time-to-event outcome. Our work shares conceptual links with the framework of predictive ability measures that aim to determine which covariates have the greatest explanatory interest. For censored data, two main frameworks have been proposed for quantifying the predictive ability of a variable to separate patients: (i) concordance, which quantifies the degree of agreement among the ranking of observed failure times according to the explained variables and is used to assess the discriminatory performance of a model [10,11]; (ii) proportion of explained variation, which quantifies the relative gain in prediction ability between a covariate-based model and a null model (without explained variables) by analogy with the well-known linear model. In this latter case, two approaches have been considered. The first one focuses on comparing empirical survival functions with and without covariates [12-15]. The second one considers statistical quantities which are directly or indirectly related to the likelihood function [16-19]. In this paper, we propose a novel index that is linked to the approach discussed above. It is related to the score statistic and well-suited for meta-selection of genomic datasets. Our index is interpreted as the ability of a gene to separate patients observed to experience the event of interest from those who do not experience the event among the risk set at every observed failure time. As shown in this study, increasing values of the index correspond to a higher effect due to the gene variable. In contrast to a test statistic, our index is not highly sensitive to sample size variation which makes it well-suited for meta-selection from datasets with various sample sizes.

We report and discuss the statistical properties of the index obtained from simulation experiments, and compare it to Allison's index [16] and its modified version [18], Nagelkerke [17] and Xu and O'Quigley's [19] indices. In addition, the properties of these indices are illustrated on a fictitious example, where data are simulated so as to mimic a real study combining datasets of different sample sizes. We then illustrate the capability of the index for combining the results of eight cancer studies of different sample sizes and with different outcomes.

## Results

### Statistical properties of the proposed index and comparison with classical indices

#### Simulation Scheme

A simulation study was performed to evaluate the behavior of the proposed index, denoted  and compare it to Allison's index [16], a modified version of Allison's index [18], Nagelkerke [17] and Xu and O'Quigley's [19] indices denoted  and  respectively (see the Methods Section for the description of the five indices) under proportional and non-proportional hazards regression models, using different values of the regression parameter, different covariate distributions and different sample sizes. Scenarios with various independent censoring distributions were also considered.

The simulation protocol was as follows. For each subject *i*, *i *= 1,⋯, *n*, we considered one covariate *Z *with either a discrete (Bernoulli *ℬ*(0.5)) or a continuous (uniform  [0, ]) distribution. These two distributions of *Z *were standardized to have the same variance. Survival times *T *were generated with the survival function *S*(*t*; *z*) = exp(-*te*^*βZ*^) (proportional hazards model) or *S*(*t*; *z*) = (1 + *t *· *e*^*βZ*^)^-1 ^(proportional odds model). For these two survival distributions, the hazard ratios were *HR *= *e*^*β *^for the proportional hazard model and *HR *= [1 + (*e*^*β *^- 1)*S*_0_(*t*)]^-1 ^for the proportional odds model, *S*_0_(*t*) refering to the baseline survivor function. In our simulation scheme, *e*^*β *^was set to 1 (null effect), to small values; 1.25, 1.5, 1.75, medium values; 2, 3, and high values; 4, 5. The sample sizes *n *of the data were taken equal to 50, 100, 500 and 1, 000.

The censoring mechanism was assumed to be independent from *T *given *Z *and the distribution of the censoring variable *C*_*i*_, *i *= 1,⋯, *n *was either uniform *C*_*i *_~ {0, *r*} or exponential *C*_*i*_~ {γ }. The calculation of the parameters *r *and *γ *as functions of the expected overall percentage of censoring *p*_*c *_is described in Additional file 1. The percentage of censoring was taken equal to 0%, 25% and 50%. For each configuration 1,000 repetitions were generated.

#### Simulation Results

The table in Additional file 2 displays the results of the simulations for  for four different sample sizes and two different covariate distributions, considering a Cox proportional hazards model. As seen from Additional file 2, when β = 0, i.e. in the absence of covariates, our index approaches 0 for *n *= 50 to 1, 000; the separability is close to 0. The index increases towards 1 with |β|, the separability increases with the effect of the covariate. When *β*≠ 0, the value of  for the different sample sizes is fairly stable, in particular for moderate or high effects (*e*^*β *^≥ 1.5). The mean values of our index for *n *= 50 to 500 are close to the mean values obtained for *n *= 1, 000 which is assumed to approach its asymptotic limit. The standard errors of  (indicated in brackets in Additional file 2) are small even when censored, and, as expected, decrease when *n *increases. Our index is slightly sensitive to the censoring rate, especially for high values of hazard ratio. Similar comments can be made when dealing with an exponential censoring mechanism (results not shown).

**Figure 1:**
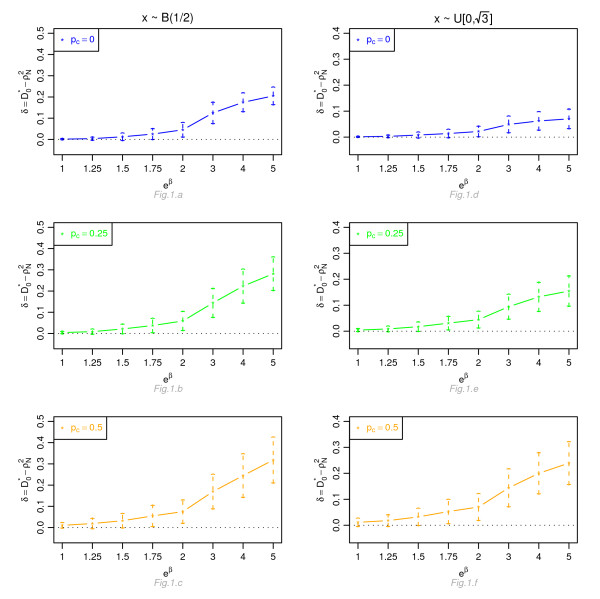
**Graphic of the differences *δ *between the mean values of  and the mean values of  as a function of the hazard ratio, for a Cox proportional hazards model**. Mean of  as a function of the relative risk *e*^*β*^, for different percentages of censoring *p*_*c*_, for a covariate with Bernoulli ℬ(1/2) or uniform  [0, ] distribution, *n *= 100 and a uniform censoring mechanism.

**Figure 2:**
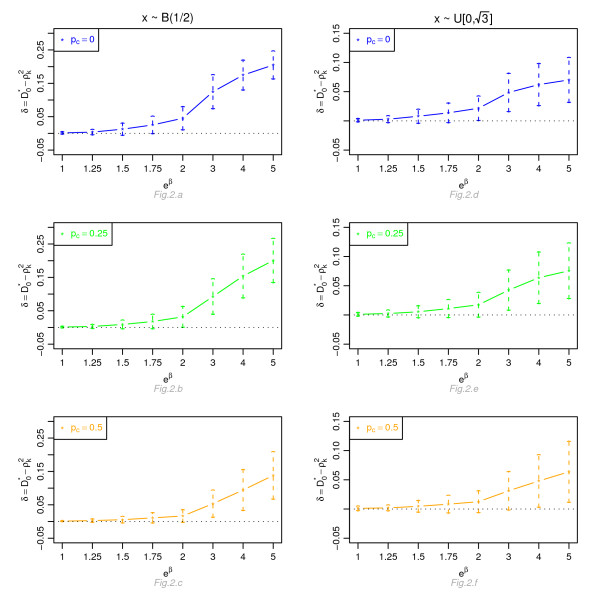
**Graphic of the differences *δ *between the mean values of  and the mean values of  as a function of the hazard ratio, for a Cox proportional hazards model**. Mean of  as a function of the relative risk *e*^*β*^, for different percentages of censoring *p*_*c*_, for a covariate with Bernoulli ℬ(1/2) or uniform  [0, ] distribution, *n *= 100 and a uniform censoring mechanism.

**Figure 3:**
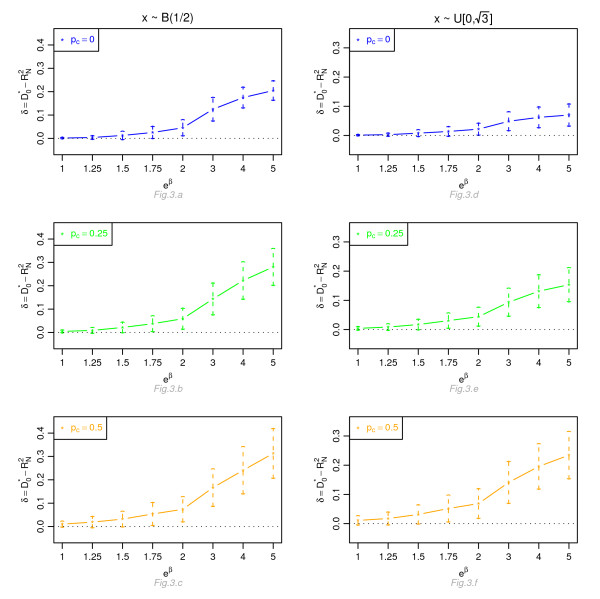
**Graphic of the differences *δ *between the mean values of  and the mean values of  as a function of the hazard ratio, for a Cox proportional hazards model**. Mean of  as a function of the relative risk *e*^*β*^, for different percentages of censoring *p*_*c*_, for a covariate with Bernoulli ℬ(1/2) or uniform  [0, ] distribution, *n *= 100 and a uniform censoring mechanism.

**Figure 4:**
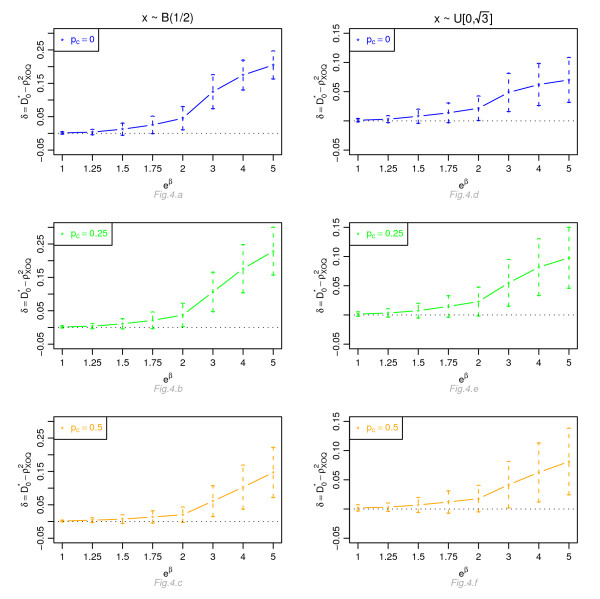
**Graphic of the differences *δ *between the mean values of  and the mean values of as a function of the hazard ratio, for a Cox proportional hazards model**. Mean of  as a function of the relative risk *e*^*β*^, for different percentages of censoring *p*_*c*_, for a covariate with Bernoulli ℬ(1/2) or uniform  [0, ] distribution, *n *= 100 and a uniform censoring mechanism.

**Figure 5:**
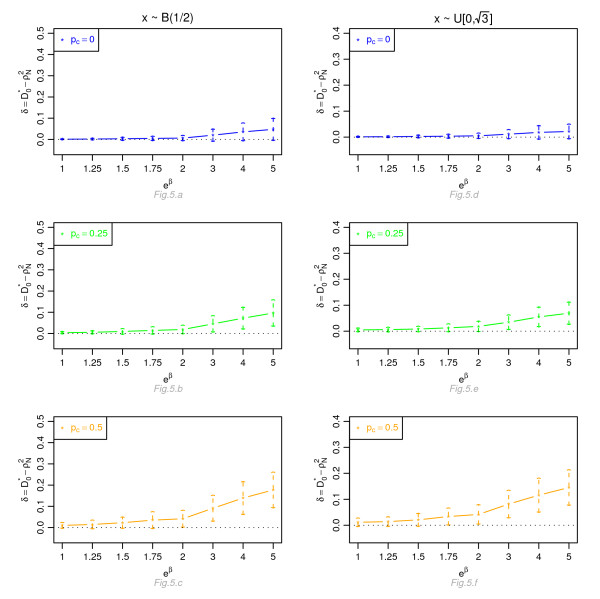
**Graphic of the differences *δ *between the mean values of  and the mean values of  as a function of the odds ratio, for a proportional odds model**. Mean of  as a function of the odds ratio *e*^*β*^, for different percentages of censoring *p*_*c*_, for a covariate with Bernoulli ℬ(1/2) or uniform  [0, ] distribution, *n *= 100 and a uniform censoring mechanism.

**Figure 6:**
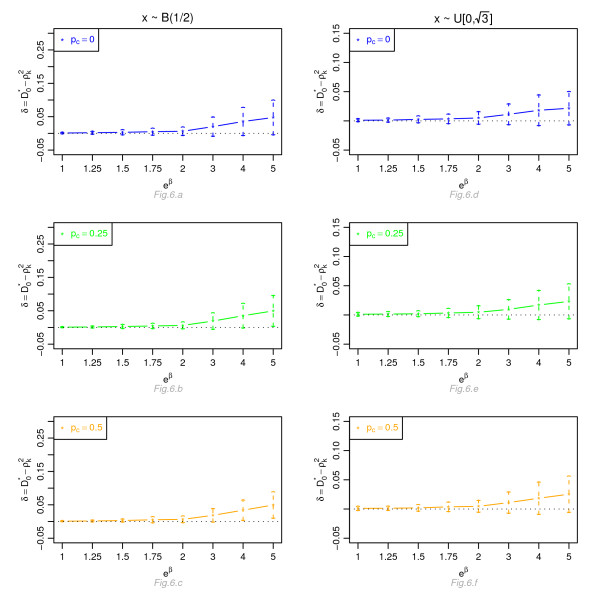
**Graphic of the differences *δ *between the mean values of and the mean values of  as a function of the odds ratio, for a proportional odds model**. Mean of  as a function of the odds ratio *e*^*β*^, for different percentages of censoring *p*_*c*_, for a covariate with Bernoulli ℬ(1/2) or uniform  [0, ] distribution, *n *= 100 and a uniform censoring mechanism.

**Figure 7:**
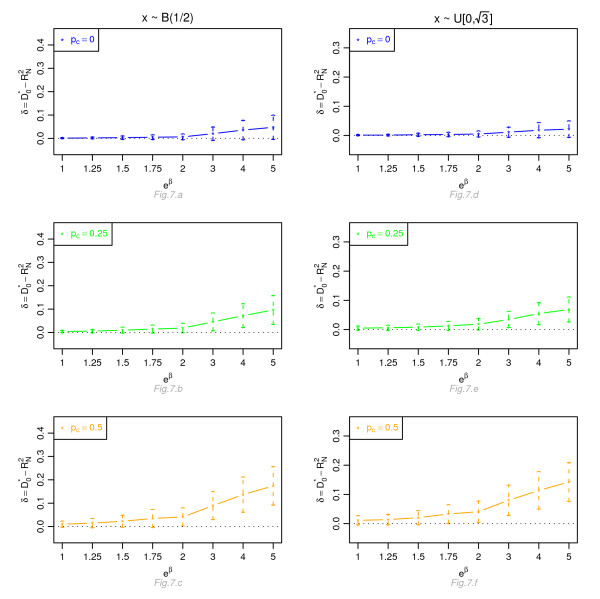
**Graphic of the differences *δ *between the mean values of  and the mean values of  as a function of the odds ratio, for a proportional odds model**. Mean of  as a function of the odds ratio *e*^*β*^, for different percentages of censoring *p*_*c*_, for a covariate with Bernoulli ℬ(1/2) or uniform  [0, ] distribution, *n *= 100 and a uniform censoring mechanism.

**Figure 8:**
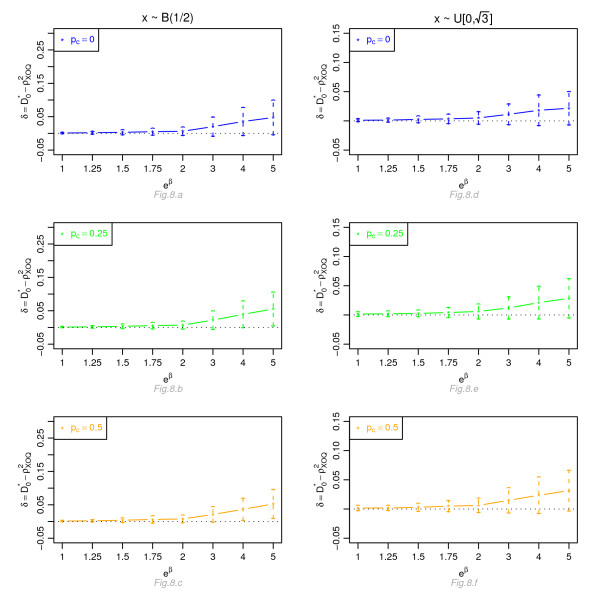
**Graphic of the differences *δ *between the mean values of  and the mean values of  as a function of the odds ratio, for a proportional odds model**. Mean of  as a function of the odds ratio *e*^*β*^, for different percentages of censoring *p*_*c*_, for a covariate with Bernoulli ℬ(1/2) or uniform  [0, ] distribution, *n *= 100 and a uniform censoring mechanism.

Figures 1, 2, 3 and 4 display, for a Cox model, the differences *δ *between the mean of  and the mean of  and  respectively, for *n *= 100, for different percentage of censoring *p*_*c*_, different covariate distributions and with a uniform censoring mechanism. The means of the differences *δ *are always positive. They are close to zero for small hazard ratios and increase with higher hazard ratios. The differences between  and  increase with the percentage of censoring, which is not surprising since the Nagelkerke's index is known to be sensitive to censoring [15]. The two indices  and  have a similar behavior relatively to . This is expected since O'Quigley *et al *[18] propose to use  as a simple working approximation of their index. The same results are obtained for *n *= 50, 500 and 1, 000 and for an exponential censoring mechanism (results not shown). For *e*^*β *^≥ 2, the 95% confidence interval for the differences of the three graphs does not comprise 0, thus in each case the difference *δ *is significant. The table in Additional file 3 and Figures 5, 6, 7 and 8 display the results of the simulations under a proportional odds model. The mean values of the different indices are lower than in the case of a proportional hazards model. All indices are more sensitive to censoring. Our index shows higher mean values than the other indices, especially in case of a Bernoulli distribution.

### Evaluation of the index in meta-selection

#### Simulation Scheme

In this subsection, using a basic example we evaluated the practical interest of our index  when combining the information contained in two studies with different sample sizes. The method used to generate the two datasets was inspired by Bair and Tibshirani [20] but modified in order to resemble the structure of real genomic data. The two datasets mimicked the analysis of the prognosis impact of transcriptional changes for a set of 1, 000 genes. The two datasets were of unequal size and composed of *n *= 150 and 50 individuals, respectively. To each individual *i*, *i *= 1,⋯, *n*; *n *= 150 or 50, we associated a survival time *T*_*i*_, a censoring time *C*_*i *_and vector of 1,000 quantitative values *Z*_*i *_= {; *g *= 1,⋯, 1, 000} (e.g. expression measurement).

To perform a fair evaluation of our index, we simulated survival data with either an exponential distribution given by *S*(*t*) = exp(-*te*^*ξ*^) (proportional hazard model) or a log-logistic survival distribution given by *S*(*t*) = (1 + *t *· *e*^*ξ*^)^-1 ^(non-proportional hazard model). For individuals *i *such as 1 ≤ *i *≤ *n*/2 (*n *= 150 or 50), the parameter *ξ *was equal to 0. For individuals *i *such as *n*/2 + 1 ≤ *i *≤ *n *(*n *= 150 or 50), *e*^*ξ *^was equal to 3 and 5. We defined individuals *i *= 1 to *n*/2 as belonging to the group of patients with low risk of occurrence of the event of interest and individuals from *i *= *n*/2 + 1 to *n *to the group with high risk of occurrence of the event.

For each dataset, censoring times *C*_*i *_(*i *= 1,⋯, *n*; *n *= 150 or 50) were considered independent from survival times and with a uniform distribution on {0, *r*}, *r *chosen in order to have an expected percentage of censoring of 30%.

The observed time to follow-up  (*i *= 1,⋯, *n*; *n *= 150 or 50) was equal to the minimum between the two previously defined times *T*_*i *_and *C*_*i*_.

For the two datasets, for each individual *i*, 1,000 gene expression values  (*i *= 1,⋯, *n*; *n *= 150 or 50; *g *= 1,⋯, 1, 000) were generated, according to the simulation scheme shown on the figure in Additional file 4. Gene expression values from *g *= 1 to 50 for individuals *i *= 1 to *n*/2 (*n *= 150 or 50) followed a log-normal distribution Log-(μ = 4, σ = 1.5) with  and . For the rest of the individuals (*i *= *n*/2 + 1,⋯, *n*; *n *= 150 or 50), gene expression values followed a distribution Log-(0, 1.5). Gene expression values from *g *= 51 to 100 for individuals *i *= 1 to *n*/2 (*n *= 150 or 50) followed a log-normal distribution with parameters μ = 3 and σ = 1.5 Log-(3, 1.5). For the rest of the individuals (*i *= *n*/2 + 1,⋯, *n*; *n *= 150 or 50), gene expression values followed a distribution Log-(0, 1.5). For gene expression values from *g *= 100 to 150 and for 40% individuals randomly selected among the *n *(*n *= 150 or 50), the  (*i *∈ *N*, *g *= 100,⋯, 150) followed a log-normal distribution Log-(1, 1.5), whereas for the remaining individuals, they followed a log-normal distribution Log-(0, 1.5). For gene expression values from *g *= 151 to 250 and for 50% individuals randomly selected among the *n*, the  (*i *∈ *N*; *g *= 151,⋯, 250) followed a log-normal distribution Log-(0.5, 1.5), whereas for the remaining individuals, they followed a log-normal distribution Log-(0, 1.5). For gene expression values from *g *= 251 to 350 and for 70% individuals randomly selected among the *n*, the  (*i *∈ *N*; *g *= 251,⋯, 350) followed a log-normal distribution Log- (0.1, 1.5), whereas for the remaining individuals, they followed a log-normal distribution Log-(0, 1.5). Finally, for gene expression values from *g *= 351 to 1, 000, the  (*i *= 1,⋯, *n*; *g *= 351,⋯, 1, 000) followed a log-normal distribution Log-(0, 1.5) for all individuals.

As genes involved in the same or related pathway are likely to be coexpressed, we introduced correlations between genes. To evaluate the behavior of our index in the context of dependent data, we generated datasets with so-called "clumpy" dependence (gene measurements are dependent in small groups, but each group is independent from the others). We applied the following protocol [21,22]. For each group of ten genes indexed by *l*, *l *= 1,⋯, 100, a random vector *A *= *a*_*il*_, *i *= 1,⋯, *n*, was generated from a standard log-normal distribution Log-(0, 1). The data matrix *Z *was then built so that  with *ρ *equal to 0.25, 0.5 or 0.75. Finally and in order to show the behavior of our index in situations close to real genomic data analysis, we standardized the dataset using classical quantile normalization [23].

In this simulation scheme, the first hundred genes were differentially expressed between the low and high risk group of patients. The other 250 genes were not linked to the low and high risk status, but were distributed differentially according to a binary factor (with various means) unlinked to the low/high risk status. The remaining genes were not linked to the low and high risk status.

For a given threshold, we calculated the number of genes common to the two simulated datasets with the five indices, for the different survival distributions, the different hazards ratio values and the different correlations between genes. We estimated the true positive fraction (TPF, number of true positives found divided by the number of truly prognostic genes) and the true negative fraction (TNF, number of true negatives divided by the number of truly non-prognostic genes) obtained with the five indices, ,  and  as a function of the threshold target value. These criteria were estimated by the mean over one hundred iterations of: (i) the proportion of correct selection (i.e. when the selected genes *g *belonged to {0,⋯, 100}) among the modified genes; (ii) the proportion of correct 'non-selection' (i.e. when the selected genes *g *belonged to {101,⋯, 1, 000}) among the non-modified genes, respectively.

Considering this procedure, the most successful criterion was the one that achieve the best operating characteristics.

#### Simulation Results

**Figure 9:**
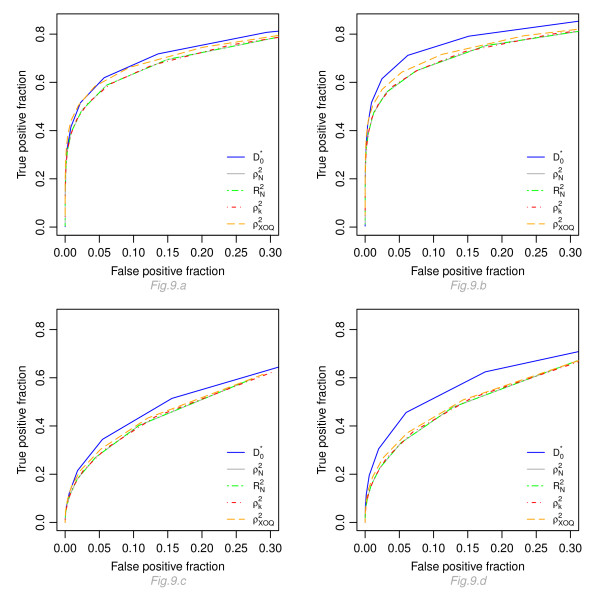
**Operating characteristics of ,  and **. Graphic of the true positive fraction versus the true negative fraction calculated for the five indices with different thresholds. Fig 9.a and 9.b display the results for a proportional hazard model, for *e*^*ξ *^= 3 and 5 respectively ; Fig 9.c and 9.d display the results for a proportional odds model, for *e*^*ξ *^= 3 and 5, respectively.

Figure 9 displays the true positive fraction versus the false negative fraction (number of false positives found divided by the number of truly non-prognostic genes) for four configurations: *ρ *= 0.5, *e*^*ξ *^= 3 and 5 and for a proportional and non-proportional model. For the five indices, higher operating characteristics are obtained under a proportional hazards model (Figure 9.a and 9.b) as compared to a proportional odds model (Fig 9.c and 9.d). Moreover, for a given distribution and a given threshold, our index gives the best results with higher true positive and true negative fractions. Results for the four other indices are very close to each other. Results with other levels of correlation (*ρ *= 0.25 and 0.75) are very close to those obtained with *ρ *= 0.5 (curves not shown here).

### Application of the index on real data

#### Datasets

In this section, we exemplify the use of the proposed index by identifying transcriptomic prognostic factors common to eight studies corresponding to five different solid tumors such as breast, lung, bladder cancer, glioma and melanoma. We compared our index to the four indices and two classical test-based criteria (q-values derived from the log-likelihood ratio and robust score statistics). The data consisted of eight independent genomic studies [24-31], with different survival outcomes and different sample sizes which samples were hybridized on a same platform (Affymetrix HU133 Plus 2.0 or HU133A ; Affymetrix, Santa Clara, CA, USA). The datasets are publicly available on the GEO site under the labels GSE2034, GSE1456, GSE11121, GSE4573, GSE5287, GSE4271, GSE4412 and GSE19234, respectively, and they are briefly described below.

##### GSE2034 cohort, breast cancer [24]

This series includes 286 lymph-node negative patients, among which 106 have developed a metastasis which is the event of interest in this study. Metastasis-free survival was defined as the time interval from treatment until the apparition of distant relapse or last follow-up. The median metastasis-free survival time was 80 months. The two years metastasis-free survival was 83.9% [79.8%; 88.3%], and the five years metastasis-free survival was 66.7% [61.4%; 72.4%].

##### GSE1456 cohort, breast cancer [25]

This series comprises 159 primary breast cancer patients (referred as Stockholm cohort). Metastasis-free survival measured the time from initial therapy until the first metastasis or last follow-up. The median metastasis-free survival time was 80 months. The two years metastasis-free survival was 87.9% [83.0%, 93.2%], and the five years metastasis-free survival was 77.6% [71.3%, 84.4%].

##### GSE11121 cohort, breast cancer [26]

This series is composed of 200 lymph node-negative breast cancer patients who were not treated by systemic therapy after surgery. Metastasis-free survival was defined as the interval from the date of therapy to the date of diagnosis of metastasis or last follow-up. The median metastasis-free survival time was 149 months. The two years metastasis-free survival was 92.9% [89.3%; 96.5%], and the five years metastasis-free survival was 85.4% [80.6%; 90.6%].

##### GSE4573 cohort, lung cancer [27]

This series comprises 129 patients with different stages of squamous cell carcinomas, who underwent surgery resection of the lung. Overall survival was defined as the time from surgery until death or last follow-up. The median overall survival time was 63 months. The two years overall survival was 70.5% [63.1%; 78.9%], and the five years overall survival was 56.8% [48.3%; 66.7%].

##### GSE5287 cohort, bladder cancer [28]

This series is composed of 30 patients who received chemotherapy. Overall survival was defined as the time from first chemotherapy to death or last follow-up. The median overall survival time was 47 months. The two years overall survival was 96.7% [90.5%; 100%], and the five years overall survival was 46.7% [31.8%; 68.4%].

##### GSE4271 cohort, glioma [29]

This study comprises 77 patients with high-grade gliomas who underwent surgery (resection) of the brain. The overall survival was measured from initial surgical resection to death or last follow-up. The median overall-survival was 21 months. The two years overall-survival was 45.5% [35.6%, 58.1%], and the five years overall-survival was 22.6% [14.7%, 34.9%].

##### GSE4412 cohort, glioma [30]

This series includes 85 patients who suffered of glioma of grade III or IV of any histologic type. The overall survival corresponded to the time from inclusion for surgical treatment to death or last follow-up. The median overall-survival was 13 months. The two years overall-survival was 33.2% [24.3%, 45.3%], and the five years overall-survival was 22.1% [12.9%, 37.7%].

##### GSE19234 cohort, melanoma [31]

The authors considered 44 metastatic melanoma tissue samples. Overall survival was referred as the time from excision of the metastatic lesion to death or last follow-up. The median overall-survival was 46 months. The two years overall-survival was 76.7% [65.0%, 90.4%], and the five years overall-survival was 56.5% [43.1%, 74%].

For these studies, the hybridizations were performed on the Affymetrix GeneChip HU133A, except for the melanoma cohort where they were performed on HU133 Plus 2.0 (HU133A+HU133B). For each patient, we considered the information obtained from 22,283 transcripts (HU133A).

For selecting a threshold target value, we considered the intersection procedure introduced by Blangiardo and Richardson [32]. The main steps of this procedure were as follows. We first ranked the genes according to a measure of interest on probability scale (e.g. the p-value or the q-value). For each experiment and for a given threshold, we counted the number of differentially expressed genes in common between the different experiments. This number was then compared to the expected number of genes in common, calculated under the hypothesis of independence between the experiments. The ratio between these two numbers was calculated for all possible thresholds. Finally, the threshold considered in the intersection selection procedure was such as the ratio was superior to 2 with a clinically relevant survival difference. Here, we used this procedure, with the following criteria: (1) our index ; (2) Allison's index ; (3) the modified version of Allison's index ; (4) Nagelkerke's index ; (5) Xu and O'Quigley's index ; (6) the q-value associated to the FDR (False Discovery Rate) calculated on the robust score statistic and estimated according to a non-parametric method [21]; (7) the q-value associated to the FDR calculated on the log-likelihood ratio statistic, estimated with the same method.

#### Selection of the Variables

The proposed index was calculated for the 22,283 gene expression measures for the eight datasets. The intersection procedure [32] led to a threshold equal of 0.07 for .

For  ≥ 0.07 (which corresponds from our simulations to a hazard ratio value around 1.5), we selected 5 transcripts related to four genes (Table 1).

**Table 1:** Top survival related genes across the eight studies, for  ≥ 0.07.

AffyID	Gene symbol	UniGene Name	Cytoband	value of HR
211596-s-at	*LRIG1*	leucine-rich repeats and immunoglobulin-like domains 1	3p14	< 1
218355-at	*KIF4A*	kinesin family member 4A	Xq13.1	> 1
218726-at	*HJURP*	Holliday junction recognition protein	2q37.1	> 1
204817-at	*ESPL1*	extra spindle pole bodies homolog 1 (S. cerevisiae)	12q13.13	> 1
38158-at	*ESPL1*	extra spindle pole bodies homolog 1 (S. cerevisiae)	12q13.13	> 1

AffyID, Affymetrix identification code for each probe set ; HR, hazard ratio

If *HR *> 1, the over-expression of the gene is associated with a worse prognosis. If *HR *< 1, the over-expression of the gene is associated with a better prognosis.

We identified *HJURP *and *LRIG1 *genes that are directly involved in tumorigenesis. *HJURP *encodes an indispensable factor for chromosomal stability in immortalized cancer cells. It is up-regulated in lung cancer [33]. *LRIG1 *encodes a protein that acts as a growth suppressor in breast cancer [34]. Its expression decreases in human breast cancer and the majority of ErbB2+ breast tumors show under-expression of *LRIG1*. In our series, the increase of *HJURP *and decrease of *LRIG1 *gene expressions are associated with a worse prognosis.

Our selection process also brought two genes involved in cell cycle regulation. Gene *KIF4A *encodes a protein critical for mitotic regulation including chromosome condensation, spindle organization and cytokinesis. It possesses a functional and physical link with the gene product of *BRCA2 *(breast cancer 2, early onset) [35]. Gene *ESPL1 *plays a central role in chromosome segregation at the onset of anaphase. Its over-expression induces aneuploidy and tumorigenesis [36]. The article of Zhang *et al *[36] showed that the *ESPL1 *transcript is over-expressed in human breast tumors. It is worth noting that *ESPL1 *and *KIF4A*, have been previously discussed in a meta-analysis conducted by Carter *et al *[37]. For these two genes, over-expression, leading to a cell proliferation, is associated with a worse prognosis.

Finally, for each gene from our selection, the hazard ratios were in the same direction in each of the eight studies.

With a same threshold of 0.07, Allison's index  selected 3 transcripts corresponding to genes *KIF4A *and *ESPL1 *and Xu and O'Quigley's index  selected 2 transcripts corresponding to gene *ESPL1*. The transcripts identified with these two indices are all included in our selected subset. For  and  with a threshold of 0.07, no transcript was selected. No transcript was selected relying on the q-value calculated with the robust score or the log-likelihood ratio statistics with a threshold of 0.40.

## Discussion

Combining heterogeneous genomic datasets to select relevant genomic factors having a common prognostic impact across various tumor entities raises some concerns regarding the choice of the statistic to be considered. In particular, the use of hypothesis testing criteria across different datasets, such as p-values or related criteria, does not seem convenient due to its sensitivity to sample size. In this paper, we propose a novel index that is well suited for a combined analysis of heterogeneous genomic datasets and which allows a selection of features with a similar prognostic impact on outcome across studies.

The index possesses the four following properties: (1) it has a straightforward and meaningful interpretation in terms of percentage of separability between patients observed to experience the event of interest and those observed not to experience the event, according to their gene expression levels. (2) It increases with the ability to separate patients according to the gene variable from 0 to 1. (3) The index is not highly dependent on the sample size. (4) It is linked to the robust score statistic derived from the partial log-likelihood which has a known asymptotic distribution, and multiple testing criteria (e.g. FDR) can easily be calculated.

Our index shares a common framework with Allison's index, its modified version, Nagelkerke and Xu and O'Quigley's indices. Indeed, these latter indices are closely related to likelihood ratio statistics whereas ours relies on the score statistic. Moreover, our index is directly interpreted in terms of separability, whereas the other indices lack intuitive interpretation.

Simulation studies show that the separability performance of our index are better than for Allison's index, its modified version, Nagelkerke and Xu and O'Quigley's indices. In our simulated example, we illustrate the good operating characteristics of our index as compared to the classical ones. However, more extensive simulations work would be necessary to evaluate its performance in various real-world scenarios.

In this work, a meta-selection performed from different solid tumors allows the identification of a small set of genes (*ESPL1*, *KIF4A*, *HJURP*, *LRIG1*) that are biologically relevant to the carcinogenesis process and show a similar ability to separate patients according to time-to-event outcomes. It would be worth conducting further studies to validate or invalidate the prognostic impact of these genes. It is important to note that for the analysis of these data we have considered a very stringent method, which relies on finding the intersection set across the different studies. If necessary, less restrictive methods can be adopted. We have to highlight that our index was primarily designed for a proportional hazard model, but, as seen from our simulations, it performs well in other contexts such as proportional odds models. This last model corresponds to frequently encountered situations where the patient population becomes more and more homogeneous as time goes on and the prognostic effect decreases with time and disappears eventually. Future studies are needed to investigate other non-proportional hazard situations.

Finally, the proposed index may be appealing for time-to-event data in other medical fields such as auto-immune and infectious diseases in which identifying prognostic factors among different entities is a promising challenge.

## Conclusion

In conclusion, we propose a novel index for identifying factors having a prognostic impact across collection of heterogeneous datasets that relies on the concept of separability and is not substantially affected by the sample size of the study. As the number of public available datasets obtained from independent studies keeps growing, our index is a promising tool which can help researchers to select a list of features of interest for further biological investigations.

## Methods

### Notations

Let  denote the value of a covariate *Z *for the *i*^*th *^subject (*i *= 1,⋯, *n*) associated to the *g*^*th *^gene (*g *= 1,⋯, *G*). For each patient *i*, let the random variables *T*_*i *_and *C*_*i *_be the survival and censoring times, which are assumed to satisfy the classical condition of independent censoring [38]. In practice, we observe  = min(*T*_*i*_, *C*_*i*_). Here we consider the possibility of the presence of ties among the uncensored failure times and we assume that there are *N *distinct times (of failure or censoring) and *k *distinct failure times (*k *≤ *N ≤ n*). For *j *= 1,⋯, *N*, let *D*(*t*_*j*_) be the set of individuals failing at time *t*_*j*_, *R*(*t*_*j*_) the risk set at *t*_*j *_and *E*(*t*_*j*_) the set of individuals failing or censored at *t*_*j*_. We denote *d*_*j*_, *n*_*j *_and *e*_*j *_the cardinals of these three sets, respectively. We also define R*(*t*_*j*_) as the risk set without the subjects failing at *t*_*j *_and R*(*t*_*l*(-*j*)_) (for *t*_*l *_<*t*_*j*_) as the risk set at time *t*_*l *_without the subjects failing or censored at *t*_*j*_. Let  be the indicator of at least one death at *t*_*j *_(where 1 is the indicator function).

The hazard function at time t for gene *g *can be written in a semi-parametric proportional hazards form as [8]

where  (*t*) is an unknown baseline hazard function, and *β*^(*g*) ^is the regression parameter to be estimated. In the presence of ties, the partial log-likelihood of the Cox model [39] can be approximated according to the Peto and Breslow method [40,41]

The first derivative of the partial log-likelihood, or score, is:

In the following, the exponent ^(*g*) ^is omitted in order to facilitate the reading. Consequently, *β *will refer to *β(*^*g)*^, *Z*_*i*_, to , ℒ to ℒ^*(g)*^and *U*_*j *_to .

### Proposed index

The proposed index is based on the interpretative property of the score deduced from the partial log-likelihood under the Cox model as recalled above. At each time *t *= *t*_*j*_, *j *= 1,⋯, *N*, we consider the quantities *U*_*j *_calculated under the null hypothesis (for *β *= 0) from the approximated Breslow partial log-likelihood

From this latter expression, it appears that, for a given covariate *Z*, and at each event time *t*_*j*_, the *U*_*j *_can be expressed as differences between the means of the covariates of the group *D*(*t*_*j*_) of patients observed to experience the event of interest, and the group R*(*t*_*j*_) of those observed to not experience the event. The *U*_*j *_provide a measure of separability between the two groups of patients *D*(*t*_*j*_) and R*(*t*_*j*_) at time *t*_*j*_. Differences close to zero indicate a weak or null separability; large differences indicate that the two groups are well separated.

Hence, a global statistic over time can be computed as the sum of these differences: . The statistic Δ_0 _is large if the two groups are well separated over time or for a few time points with large values but with the same directional effect (proportional hazard assumption).

For distributional reasons which will appear later, instead of the *U*_*j*_, *j *= 1,⋯, *N*, we use closely related quantities *W*_*j *_derived from the paper by Lin and Wei [42]. In the presence of ties, we propose the following formula for *W*_*j*_

The term  is a weighted average of the score calculated at times *t*_*l *_prior to time *t*_*j *_(*t*_*l *_<*t*_*j*_). The sum of the so-called "robust" *W*_*j*_, *j *= 1,⋯, *N *is identical to the sum of the *U*_*j*_, but, as shown by Lin and Wei, the *W*_*j *_are independent and identically distributed, while the *U*_*j *_are not. Simple calculations show that the *W*_*j *_can be rearranged as in the following expression:

with

The usual global robust score is computed as the sum of the differences *W*_*j*_, *j *= 1,⋯, *N *(which is also equal to the sum of the *U*_*j*_). So, Δ_0 _can be re-expressed as the sum of the *W*_*j*_:

In Additional file 5, we show that  ranges from 0 (null separability under the proportional hazard model) to  (maximal separability). The value **D**_*max *_is a theoretical maximum of **D**_0_, which corresponds to the case where *β *tends to infinity.

Finally,

gives a meaningful index that can be interpreted as the percentage of separability over time between the event/non-event groups. It is equal to 0 in the absence of separability and increases toward 1 as the separability rises. To a factor *k*, the index can also be interpreted as the robust score statistic (**S**_0 _= *k *· ) [43], whose distribution under the null hypothesis is an asymptotic chi-square distribution with 1 degree of freedom. Multiple error criteria can thus be computed using a parametric or non-parametric approach.

### Existing indices

Several indices of predictive accuracy have been proposed in the literature. Here, only indices with direct or indirect links to the likelihood ratio function and with a known distribution after transformation under the null hypothesis are considered.

The indices are the following: (i) Allison's index [16], based on a transformation of the partial log-likelihood ratio test; (ii) a modified version of Allison's index  proposed by O'Quigley *et al *[18]; (iii) Nagelkerke's index [17], which is a modification of Allison's index dividing it by its maximum value, and (iv) Xu and O'Quigley's index [19] based on a transformation of the Kullback-Leibler distance between the null and the alternative models.

The expressions of these four indices for one given gene *g*; *g *= 1,⋯, *G *are reminded here:

**(i) **Allison's index:

**(ii) **Modified version of Allison's index:

In this version of the index, the log-likelihood ratio is divided by the number of failures *k*. As discussed by O'Quigley *et al *[18], the original version is more sensitive to censorship than the modified one. In particular, O'Quigley *et al *show that  approaches 0 as the percentage of censored observation approaches 100%.

**(iii) **Nagelkerke's index:

with

This index was initially proposed to fully exploit the range [0, 1], which is not the case with the original version of Allison's index.

**(iv) **Xu and O'Quigley's index:

with

where  and  is the Kaplan-Meier estimator of the distribution function of *T*.

The term  is derived from twice the Kullback-Leibler distance between the null model (*β *= 0) and the model taking the covariates into account (*β *≠ 0).

The conditional probability  that the individual indexed by *i *is selected for failure at the time *t*_*j *_is given by

## Authors' contributions

SR, TM and PB developed the original index. PB coordinated the project and is SR's PhD thesis advisor. All authors read and approved the final manuscript.

## Supplementary Material

Additional file 1 **Calculation of the parameters of the different censoring mechanisms**. We explain the procedure adopted for the calculation of the parameters of uniform and exponential censoring mechanisms as functions of the distribution of the covariates and the percentage of censoring. Click here for file

Additional file 2**Mean values of  in the framework of a Cox proportional hazards model, for different relative risks *e*^*β*^, different percentages of censoring *p*_*c *_and different sample sizes *n*, calculated for a covariate with Bernoulli ℬ(1/2) or a uniform  [0, ] distribution, for a uniform censoring mechanism (1,000 repetitions). The standard errors are indicated in brackets**. Table with the mean values of  in the framework of a Cox proportional hazards model for different configurations.Click here for file

Additional file 3**Mean values of  in the framework of a proportional odds model, for different odds ratios *e*^*β*^, different percentages of censoring *p*_*c *_and different sample sizes *n*, calculated for a covariate with Bernoulli ℬ(1/2) or a uniform  [0, ] distribution, for a uniform censoring mechanism (1,000 repetitions).** The standard errors are indicated in brackets. Table with the mean values of  in the framework of a proportional odds model for different configurations.Click here for file

Additional file 4 **Representation of the simulated example**. Simple representation of the simulation plan. Click here for file

Additional file 5**Proof establishing that  ranges from 0 to **. We show that .Click here for file
