# Supplementary material for: Identifying common prognostic factors in genomic cancer studies: A novel index for censored outcomes
Source: BMC Bioinformatics. 2010 Mar 24;11:150. doi: 10.1186/1471-2105-11-150 (PMC2863163; doi:10.1186/1471-2105-11-150)
Supplement: Additional file 1 — Calculation of the parameters of the different censoring mechanisms. We explain the procedure adopted for the calculation of the parameters of uniform and exponential censoring mechanisms as functions of the distribution of the covariates and the percentage of censoring. [file 1471-2105-11-150-S1.PDF]

**Additional file 1 — Calculation of the parameters of the different censoring mechanisms.**

The censoring mechanism was assumed to be independent uniform $C_i \sim \mathcal{U}\{0, r\}$ or exponential $C_i \sim \mathcal{E}(\gamma)$.

• For a Cox proportional hazards model, the survival function was exponential with parameters $\lambda = \lambda_0 e^{\beta Z}$. In order to determine $r$ and $\gamma$ for a given value of the expected overall percentage $p_c$ of censoring, we solved iteratively for $r_p$ and $\gamma_p$ (value of $r$ and $\gamma$ for a given percentage of censoring $p_c$, respectively) in the following equations.

Under the null hypothesis ($\beta = 0$), the equations to solve were

$$p_c = \frac{1}{\lambda_0 r_p}\left(1 - e^{-\lambda_0 r_p}\right) \text{ , for a uniform censoring}$$

and

$$p_c = \frac{\gamma_p}{\lambda_0 + \gamma_p} \text{ , for an exponential censoring}$$

Under the alternative hypothesis (i.e. with covariate, $\beta \neq 0$), the equations to solve were

$$p_c = \int_{\mathcal{R}} \left\{ \frac{1}{r_p \lambda_0 \exp(\beta z)} \left(1 - e^{-\lambda_0 r_p \exp(\beta z)}\right) f_Z(z) \right\} dz$$

for a uniform censoring, and

$$p_c = \int_{\mathcal{R}} \left\{ \frac{\gamma_p}{\gamma_p + \lambda_0 \exp(\beta z)} f_Z(z) \right\} dz$$

for an exponential censoring. The function $f_Z(z)$ denote the density of the covariate $Z$.

• Under a proportional odds model, $T$ had a log-logistic distribution with density $f(t) = \lambda_0 e^{-\beta z}/(1 + te^{-\beta z})^2$. In this case, the equations to solve under the null hypothesis were

$$p_c = \frac{\ln(r)}{r} \text{ , for a uniform censoring}$$

and

$$p_c = \int_0^\infty \frac{\gamma e^{-\gamma c}}{1 + c} dc \text{ , for an exponential censoring}$$

Under the alternative hypothesis, the equations were

$$p_c = \int_{\mathcal{R}} \left\{ \frac{\ln(1 + re^{-\beta z})}{r} f_Z(z) \right\} dz$$

for a uniform censoring, and

$$p_c = \int_{\mathcal{R}} \left\{ \int_0^\infty \left( \frac{\gamma e^{-\gamma c}}{1 + ce^{-\beta z}} \right) dc \right\} f_Z(z) dz$$

for an exponential censoring.

• For both models, for one subject $i$ and for a uniform censoring, two independent uniform variates $U_{0.25}^{i}$ and $U_{0.5}^{i}$ on $\{0, r_{0.25}^{i}\}$ and $\{0, r_{0.5}^{i}\}$ were generated, respectively. For an exponential censoring, two independent exponential variates $\mathcal{E}_{0.25}^{i}$ and $\mathcal{E}_{0.5}^{i}$ with parameters $\gamma_{0.25}^{i}$ and $\gamma_{0.5}^{i}$ were generated, respectively. In both cases, the three variates $T^{i}$, $\min(T^{i}, C_{0.25}^{i})$ and $\min(T^{i}, C_{0.5}^{i})$, with $C^{i} = U^{i}$ or $\mathcal{E}^{i}$, represent the observed times for the subject $i$ in three situations with an expected percentage of censoring equal to 0, 25 and 50%, respectively.
