# Supplementary material for: Identifying common prognostic factors in genomic cancer studies: A novel index for censored outcomes
Source: BMC Bioinformatics. 2010 Mar 24;11:150. doi: 10.1186/1471-2105-11-150 (PMC2863163; doi:10.1186/1471-2105-11-150)
Supplement: Additional file 2 — Mean values of in the framework of a Cox proportional hazards model, for different relative risks eβ, different percentages of censoring pc and different sample sizes n, calculated for a covariate with Bernoulli ℬ(1/2) or a uniform [0, ] distribution, for a uniform censoring mechanism (1,000 repetitions). The standard errors are indicated in brackets. Table with the mean values of in the framework of a Cox proportional hazards model for different configurations. [file 1471-2105-11-150-S2.PDF]

Additional file 2 - Mean values of $\mathbf{D}_0^*$ in the framework of a Cox proportional hazards model, for different relative risks e^β, different percentages of censoring p_c and different sample sizes n, calculated for a covariate with Bernoulli $\mathcal{B}(1/2)$ or a uniform $\mathcal{U}[0,\sqrt{3}]$ distribution, for a uniform censoring mechanism (1,000 repetitions). The standard errors are indicated in brackets.

		$Z \sim \mathcal{B}(1/2)$				$Z \sim \mathcal{U}[0,\sqrt{3}]$			
e^β	p_c	$\mathbf{D}_0^*(n=50)$	$\mathbf{D}_0^*(n=100)$	$\mathbf{D}_0^*(n=500)$	$\mathbf{D}_0^*(n=1000)$	$\mathbf{D}_0^*(n=50)$	$\mathbf{D}_0^*(n=100)$	$\mathbf{D}_0^*(n=500)$	$\mathbf{D}_0^*(n=1000)$
1	0	0.0257(0.0431)	0.0114(0.0180)	0.0021(0.0031)	0.0010(0.0015)	0.0243(0.0349)	0.0115(0.0159)	0.0021(0.0032)	0.0011(0.0015)
	0.25	0.0314(0.0456)	0.0144(0.0211)	0.0026(0.0039)	0.0014(0.0020)	0.0294(0.0439)	0.0135(0.0199)	0.0028(0.0040)	0.0013(0.0019)
	0.50	0.0431(0.0620)	0.0199(0.0262)	0.0041(0.0057)	0.0020(0.0027)	0.0440(0.0591)	0.0212(0.0308)	0.0040(0.0061)	0.0021(0.0027)
1.25	0	0.0394(0.0550)	0.0236(0.0313)	0.0151(0.0111)	0.0142(0.0078)	0.0368(0.0457)	0.0241(0.0276)	0.0154(0.0114)	0.0140(0.0073)
	0.25	0.0474(0.0688)	0.0265(0.0342)	0.0154(0.0136)	0.0139(0.0083)	0.0427(0.0576)	0.0277(0.0334)	0.0152(0.0124)	0.0137(0.0079)
	0.50	0.0556(0.0760)	0.0359(0.0465)	0.0156(0.0148)	0.0138(0.0101)	0.0562(0.0752)	0.0339(0.0435)	0.0172(0.0158)	0.0137(0.0099)
1.5	0	0.0696(0.0758)	0.0583(0.0557)	0.0474(0.0225)	0.0468(0.0149)	0.0659(0.0666)	0.0558(0.0472)	0.0442(0.0195)	0.0435(0.0138)
	0.25	0.0772(0.0926)	0.0604(0.0571)	0.0443(0.0221)	0.0438(0.0163)	0.0702(0.0767)	0.0533(0.0511)	0.0438(0.0215)	0.0418(0.0153)
	0.50	0.0865(0.1084)	0.0609(0.0641)	0.0441(0.0253)	0.0425(0.0179)	0.0831(0.0948)	0.0605(0.0618)	0.0444(0.0254)	0.0416(0.0173)
1.75	0	0.1169(0.1072)	0.0992(0.0694)	0.0919(0.0324)	0.0903(0.0224)	0.1033(0.0842)	0.0897(0.0598)	0.0808(0.0263)	0.0819(0.0190)
	0.25	0.1177(0.1159)	0.0996(0.0772)	0.0867(0.0331)	0.0849(0.0236)	0.1065(0.0983)	0.0921(0.0684)	0.0802(0.0307)	0.0794(0.0209)
	0.50	0.1268(0.1276)	0.1011(0.0892)	0.0817(0.0362)	0.0802(0.0247)	0.1163(0.1153)	0.1001(0.0865)	0.0804(0.0348)	0.0788(0.0245)
2	0	0.1612(0.1229)	0.1536(0.0854)	0.1414(0.0408)	0.1425(0.0280)	0.1344(0.0937)	0.1267(0.0669)	0.1222(0.0314)	0.1215(0.0227)
	0.25	0.1635(0.1400)	0.1467(0.0939)	0.1320(0.0433)	0.1320(0.0296)	0.1475(0.1242)	0.1310(0.0796)	0.1209(0.0356)	0.1202(0.0267)
	0.50	0.1639(0.1500)	0.1383(0.0977)	0.1235(0.0454)	0.1203(0.0306)	0.1546(0.1368)	0.1332(0.0962)	0.1193(0.0415)	0.1161(0.0291)
3	0	0.3338(0.1563)	0.3427(0.1165)	0.3377(0.0526)	0.3396(0.0365)	0.2712(0.1278)	0.2604(0.0884)	0.2624(0.0406)	0.2618(0.0287)
	0.25	0.3390(0.1903)	0.3227(0.1296)	0.3294(0.0612)	0.3261(0.0457)	0.2798(0.1485)	0.2718(0.1068)	0.2697(0.0486)	0.2700(0.0354)
	0.50	0.3232(0.2080)	0.3052(0.1394)	0.2866(0.0659)	0.2858(0.0460)	0.2922(0.1883)	0.2752(0.1318)	0.2626(0.0565)	0.2646(0.0394)
4	0	0.4505(0.1427)	0.4653(0.1085)	0.4766(0.0476)	0.4796(0.0363)	0.3526(0.1226)	0.3544(0.0910)	0.3585(0.0416)	0.3597(0.0282)
	0.25	0.4721(0.1847)	0.4767(0.1460)	0.4915(0.0660)	0.4868(0.0441)	0.3863(0.1637)	0.3795(0.1147)	0.3803(0.0508)	0.3835(0.0367)
	0.50	0.4654(0.2380)	0.4349(0.1711)	0.4366(0.0742)	0.4308(0.0513)	0.4072(0.2012)	0.3900(0.1391)	0.3838(0.0633)	0.3810(0.0466)
5	0	0.5327(0.1439)	0.5495(0.0987)	0.5656(0.0433)	0.5665(0.0302)	0.4169(0.1214)	0.4201(0.0854)	0.4274(0.0382)	0.4259(0.0284)
	0.25	0.5832(0.1930)	0.5927(0.1430)	0.6057(0.0627)	0.6094(0.0458)	0.4673(0.1674)	0.4604(0.1156)	0.4643(0.0500)	0.4640(0.0371)
	0.50	0.5702(0.2363)	0.5610(0.1762)	0.5578(0.0793)	0.5514(0.0546)	0.4928(0.1925)	0.4726(0.1451)	0.4721(0.0649)	0.4714(0.0470)
