# Supplementary material for: Identifying common prognostic factors in genomic cancer studies: A novel index for censored outcomes
Source: BMC Bioinformatics. 2010 Mar 24;11:150. doi: 10.1186/1471-2105-11-150 (PMC2863163; doi:10.1186/1471-2105-11-150)
Supplement: Additional file 3 — Mean values of in the framework of a proportional odds model, for different odds ratios eβ, different percentages of censoring pc and different sample sizes n, calculated for a covariate with Bernoulli ℬ(1/2) or a uniform [0, ] distribution, for a uniform censoring mechanism (1,000 repetitions). The standard errors are indicated in brackets. Table with the mean values of in the framework of a proportional odds model for different configurations. [file 1471-2105-11-150-S3.PDF]

Additional file 3 - Mean values of $\mathbf{D}_0^*$ in the framework of a proportional odds model, for different odds ratios e^β, different percentages of censoring p_c and different sample sizes n, calculated for a covariate with Bernoulli $\mathcal{B}(1/2)$ or a uniform $\mathcal{U}[0,\sqrt{3}]$ distribution, for a uniform censoring mechanism (1,000 repetitions). The standard errors are indicated in brackets.

		$Z \sim \mathcal{B}(1/2)$				$Z \sim \mathcal{U}[0,\sqrt{3}]$			
e^β	p_c	$\mathbf{D}_0^*(n=50)$	$\mathbf{D}_0^*(n=100)$	$\mathbf{D}_0^*(n=500)$	$\mathbf{D}_0^*(n=1000)$	$\mathbf{D}_0^*(n=50)$	$\mathbf{D}_0^*(n=100)$	$\mathbf{D}_0^*(n=500)$	$\mathbf{D}_0^*(n=1000)$
1	0	0.0275(0.0412)	0.0099(0.0153)	0.0021(0.0029)	0.0010(0.0014)	0.0225(0.0307)	0.0110(0.0152)	0.0020(0.0028)	0.0010(0.0014)
	0.25	0.0291(0.0441)	0.0137(0.0196)	0.0027(0.0038)	0.0013(0.0019)	0.0305(0.0420)	0.0159(0.0235)	0.0028(0.0038)	0.0014(0.0020)
	0.50	0.0417(0.0566)	0.0198(0.0277)	0.0042(0.0059)	0.0021(0.0028)	0.0432(0.0596)	0.0216(0.0312)	0.0040(0.0060)	0.0021(0.0032)
1.25	0	0.0269(0.0435)	0.0147(0.0229)	0.0052(0.0058)	0.0042(0.0040)	0.0265(0.0363)	0.0133(0.0201)	0.0052(0.0057)	0.0041(0.0038)
	0.25	0.0337(0.0463)	0.0189(0.0250)	0.0071(0.0083)	0.0061(0.0058)	0.0373(0.0544)	0.0199(0.0279)	0.0071(0.0079)	0.0057(0.0052)
	0.50	0.0505(0.0674)	0.0279(0.0393)	0.0104(0.0115)	0.0086(0.0076)	0.0524(0.0700)	0.0268(0.0360)	0.0101(0.0110)	0.0076(0.0070)
1.5	0	0.0372(0.0560)	0.0231(0.0309)	0.0131(0.0107)	0.0114(0.0071)	0.0351(0.0486)	0.0227(0.0290)	0.0129(0.0100)	0.0119(0.0069)
	0.25	0.0443(0.0620)	0.0310(0.0386)	0.0178(0.0136)	0.0164(0.0091)	0.0477(0.0620)	0.0274(0.0330)	0.0174(0.0133)	0.0163(0.0093)
	0.50	0.0620(0.0846)	0.0437(0.0515)	0.0243(0.0182)	0.0221(0.0124)	0.0688(0.0901)	0.0404(0.0483)	0.0246(0.0189)	0.0227(0.0130)
1.75	0	0.0480(0.0630)	0.0340(0.0388)	0.0228(0.0143)	0.0213(0.0096)	0.0480(0.0596)	0.0314(0.0365)	0.0222(0.0143)	0.0208(0.0094)
	0.25	0.0610(0.0792)	0.0448(0.0471)	0.0314(0.0182)	0.0301(0.0125)	0.0602(0.0731)	0.0427(0.0446)	0.0303(0.0179)	0.0295(0.0124)
	0.50	0.0795(0.0954)	0.0659(0.0723)	0.0434(0.0253)	0.0415(0.0174)	0.0755(0.0880)	0.0645(0.0631)	0.0444(0.0254)	0.0405(0.0177)
2	0	0.0600(0.0776)	0.0431(0.0446)	0.0338(0.0189)	0.0314(0.0118)	0.0548(0.0644)	0.0431(0.0423)	0.0319(0.0166)	0.0314(0.0123)
	0.25	0.0791(0.0946)	0.0587(0.0550)	0.0481(0.0225)	0.0440(0.0160)	0.0738(0.0819)	0.0601(0.0566)	0.0460(0.0211)	0.0440(0.0160)
	0.50	0.1003(0.1131)	0.0783(0.0743)	0.0654(0.0324)	0.0624(0.0225)	0.0976(0.1095)	0.0807(0.0711)	0.0629(0.0306)	0.0609(0.0209)
3	0	0.1176(0.1124)	0.0998(0.0781)	0.0840(0.0330)	0.0803(0.0219)	0.1036(0.0898)	0.0912(0.0642)	0.0779(0.0292)	0.0750(0.0193)
	0.25	0.1536(0.1320)	0.1290(0.0885)	0.1135(0.0376)	0.1114(0.0259)	0.1381(0.1159)	0.1142(0.0744)	0.1058(0.0338)	0.1053(0.0231)
	0.50	0.1884(0.1591)	0.1698(0.1098)	0.1539(0.0471)	0.1495(0.0333)	0.1697(0.1492)	0.1595(0.1012)	0.1427(0.0427)	0.1420(0.0316)
4	0	0.1678(0.1341)	0.1517(0.0991)	0.1299(0.0419)	0.1274(0.0305)	0.1506(0.1138)	0.1347(0.0842)	0.1168(0.0357)	0.1164(0.0253)
	0.25	0.2139(0.1638)	0.1953(0.1087)	0.1787(0.0466)	0.1732(0.0333)	0.1874(0.1328)	0.1779(0.0950)	0.1603(0.0401)	0.1575(0.0296)
	0.50	0.2648(0.1813)	0.2560(0.1344)	0.2331(0.0572)	0.2269(0.0389)	0.2509(0.1702)	0.2270(0.1207)	0.2147(0.0530)	0.2117(0.0369)
5	0	0.2190(0.1577)	0.1922(0.1152)	0.1758(0.0511)	0.1696(0.0342)	0.1859(0.1220)	0.1685(0.0886)	0.1543(0.0406)	0.1512(0.0287)
	0.25	0.2779(0.1818)	0.2518(0.1227)	0.2369(0.0538)	0.2307(0.0381)	0.2476(0.1554)	0.2228(0.0998)	0.2079(0.0456)	0.2043(0.0329)
	0.50	0.3427(0.2007)	0.3247(0.1457)	0.2999(0.0645)	0.2967(0.0437)	0.2982(0.1817)	0.2886(0.1259)	0.2710(0.0566)	0.2715(0.0412)
