# Supplementary material for: Identifying common prognostic factors in genomic cancer studies: A novel index for censored outcomes
Source: BMC Bioinformatics. 2010 Mar 24;11:150. doi: 10.1186/1471-2105-11-150 (PMC2863163; doi:10.1186/1471-2105-11-150)
Supplement: Additional file 4 — Representation of the simulated example. Simple representation of the simulation plan. [file 1471-2105-11-150-S4.PDF]

Additional file 4 — Representation of the simulated example.

		$n = 50$ or 150 patients	
		Low risk patients group $(n/2)$	*High risk patients group* $(n/2)$
Survival time distribution		$S(t) = t$ or $S(t) = [1 + t]^{-1}$	$S(t) = te^{-\xi}$ or $S(t) = [1 + t \cdot e^{\xi}]^{-1}$
Genes G	$g = 1 - 50$	Log-$\mathcal{N}(4, 1.5)$	Log-$\mathcal{N}(0, 1.5)$
	$g = 51 - 100$	Log-$\mathcal{N}(3, 1.5)$	Log-$\mathcal{N}(0, 1.5)$
	$g = 101 - 150$	60% Log-$\mathcal{N}(0, 1.5)$ + 40% Log-$\mathcal{N}(1, 1.5)$	
	$g = 151 - 250$	50% Log-$\mathcal{N}(0, 1.5)$ + 50% Log-$\mathcal{N}(0.5, 1.5)$	
	$g = 251 - 350$	30% Log-$\mathcal{N}(0, 1.5)$ + 70% Log-$\mathcal{N}(0.1, 1.5)$	
	$g = 301 - 1000$	Log-$\mathcal{N}(0, 1.5)$	

To complete this simulation scheme, correlations were added for each group of 10 genes ($\rho = 0.25; 0.5$ or 0.75). A quantile normalization was finally applied.
