# Supplementary material for: Identifying common prognostic factors in genomic cancer studies: A novel index for censored outcomes
Source: BMC Bioinformatics. 2010 Mar 24;11:150. doi: 10.1186/1471-2105-11-150 (PMC2863163; doi:10.1186/1471-2105-11-150)
Supplement: Additional file 5 — Proof establishing that ranges from 0 to . We show that . [file 1471-2105-11-150-S5.PDF]

Additional file 5 — Proof establishing that $\mathbf{D}_0 = \left(\sum_{j=1}^{N} U_j\right)^2/k = \left(\sum_{j=1}^{N} W_j\right)^2/k$ ranges from 0 to $\mathbf{D}_{max} = \sum_{j=1}^{N} W_j^2$.

(i) First, we can easily show that the sum of the W_j is equal to the sum of the U_j over time, i.e. that $\sum_{j=1}^{N} W_j = \sum_{j=1}^{N} U_j$. This amounts to show that $\sum_{j=1}^{N} \widehat{E}U_j = 0$.

The expression of this latter quantity in the absence of ties ($N = n$) for a proportional hazards model is the following

$$\sum_{j=1}^{n} \widehat{E}U_j = \sum_{j=1}^{n} \left[\sum_{l=1}^{j} \frac{\eta_l}{n_l} \left(Z_j - \frac{1}{n_l} \sum_{r \in R(t_l)} Z_r \right) \right]$$

The first term of this difference can be developed as

$$\begin{aligned}
\sum_{j=1}^{n} \sum_{l=1}^{j} \frac{\eta_l}{n_l} Z_j &= \sum_{j=1}^{n} \left(\frac{\eta_1}{n_1} Z_j + \frac{\eta_2}{n_2} Z_j + \cdots + \frac{\eta_j}{n_j} Z_j \right) \\
&= Z_1 \left(\frac{\eta_1}{n_1} \right) + Z_2 \left(\frac{\eta_1}{n_1} + \frac{\eta_2}{n_2} \right) + \cdots + Z_n \left(\frac{\eta_1}{n_1} + \frac{\eta_2}{n_2} + \cdots + \frac{\eta_n}{n_n} \right)
\end{aligned}$$

The second term can also be developed

$$\begin{aligned}
\sum_{j=1}^{n} \sum_{l=1}^{j} \frac{\eta_l}{n_l^2} \sum_{r=l}^{n} Z_r &= \sum_{j=1}^{n} \sum_{l=1}^{j} \frac{\eta_l}{n_l^2} \left(Z_l + \cdots + Z_n \right) \\
&= \sum_{j=1}^{n} \left[\frac{\eta_1}{n_1^2} \left(Z_1 + \cdots + Z_n \right) + \frac{\eta_2}{n_2^2} \left(Z_2 + \cdots + Z_n \right) + \cdots + \frac{\eta_j}{n_j^2} \left(Z_j + \cdots + Z_n \right) \right] \\
&= n_1 \times \frac{\eta_1}{n_1^2} \left(Z_1 + \cdots + Z_n \right) + n_2 \times \frac{\eta_2}{n_2^2} \left(Z_2 + \cdots + Z_n \right) + \cdots + n_n \times \frac{\eta_n}{n_n^2} Z_n \\
&= \frac{\eta_1}{n_1} \left(Z_1 + \cdots + Z_n \right) + \frac{\eta_2}{n_2} \left(Z_2 + \cdots + Z_n \right) + \cdots + \frac{\eta_n}{n_n} Z_n \\
&= Z_1 \left(\frac{\eta_1}{n_1} \right) + Z_2 \left(\frac{\eta_1}{n_1} + \frac{\eta_2}{n_2} \right) + \cdots + Z_n \left(\frac{\eta_1}{n_1} + \frac{\eta_2}{n_2} + \cdots + \frac{\eta_n}{n_n} \right)
\end{aligned}$$

We can see that

$$\sum_{j=1}^{n} \sum_{l=1}^{j} \frac{\eta_l}{n_l^2} \sum_{r \in R(t_l)} Z_r = \sum_{j=1}^{n} \sum_{l=1}^{j} \frac{\eta_l}{n_l} Z_j$$

Thus $\sum_{j=1}^{n} \widehat{E}U_j = 0$, which implies that $\sum_{j=1}^{n} W_j = \sum_{j=1}^{n} U_j$.

In the presence of tied observations, the proof can be obtained in the same but more burdensome way.

The property given above implies that $\left(\sum_{j=1}^{N} W_j\right)^2 = \left(\sum_{j=1}^{N} U_j\right)^2$. The sum of the W_j, as well as the sum of the U_j is thus composed of k terms (where k is the number of failure times).

(ii) Second, as a consequence of (i), the sum $\sum_{j=1}^{N} \left(W_j - \sum_{l=1}^{N} \frac{W_l}{k} \right)$ is composed of k terms. This allows to

write the following equalities:

$$\begin{aligned}
\sum_{j=1}^{N}\left(W_j - \sum_{l=1}^{N}\frac{W_l}{k}\right)^2 &= \sum_{j=1}^{N} W_j^2 - 2\sum_{j=1}^{N} W_j \sum_{l=1}^{N}\frac{W_l}{k} + \sum_{j=1}^{N}\left(\sum_{l=1}^{N}\frac{W_l}{k}\right)^2 \\
&= \sum_{j=1}^{N} W_j^2 - 2\frac{\left(\sum_{j=1}^{N} W_j\right)^2}{k} + \frac{\left(\sum_{j=1}^{N} W_j\right)^2}{k} \\
&= \sum_{j=1}^{N} W_j^2 - \left(\sum_{j=1}^{N} W_j\right)^2 /k
\end{aligned}$$

As the sum of squares $\sum_{j=1}^{N}\left(W_j - \sum_{l=1}^{N}\frac{W_l}{k}\right)^2$ is positive, we have $\sum_{j=1}^{N} W_j^2 \geq \left(\sum_{j=1}^{N} W_j\right)^2 /k$. It is now straightforward to see that $\left(\sum_{j=1}^{N} W_j\right)^2 /k$ is comprised between 0 and $\mathbf{D}_{max} = \sum_{j=1}^{N} W_j^2$.
